# Effect of Integrated Water-Nutrient Management Strategies on Soil Erosion Mediated Nutrient Loss and Crop Productivity in Cabo Verde Drylands

**DOI:** 10.1371/journal.pone.0134244

**Published:** 2015-07-31

**Authors:** Isaurinda Baptista, Coen Ritsema, Violette Geissen

**Affiliations:** 1 Instituto Nacional de Investigação e Desenvolvimento Agrário, INIDA, Praia, Cabo Verde; 2 Soil Physics and Sustainable Land Management group (SLM), Wageningen University (WUR), Wageningen, The Netherlands; 3 INRES, University of Bonn, Bonn, Germany; Banaras Hindu University, INDIA

## Abstract

Soil erosion, runoff and related nutrient losses are a big risk for soil fertility in Cabo Verde drylands. In 2012, field trials were conducted in two agro-ecological zones to evaluate the effects of selected techniques of soil-water management combined with organic amendments (T1: compost/manure + soil surfactant; T2: compost/animal or green manure + pigeon-pea hedges + soil surfactant; T3: compost/animal or green manure + mulch + pigeon-pea hedges) on nitrogen (N) and phosphorus (P) losses in eroded soil and runoff and on crop yields. Three treatments and one control (traditional practice) were tested in field plots at three sites with a local maize variety and two types of beans. Runoff and eroded soil were collected after each erosive rain, quantified, and analysed for NO_3_-N and PO_4_-P concentrations. In all treatments runoff had higher concentrations of NO_3_-N (2.20-4.83 mg L^-1^) than of PO_4_-P (0.02-0.07 mg L^-1^), and the eroded soil had higher content of PO_4_-P (5.27-18.8 mg g^-1^) than of NO_3_-N (1.30-8.51 mg g^-1^). The control had significantly higher losses of both NO_3_-N (5.4, 4.4 and 19 kg ha^-1^) and PO_4_-P (0.2, 0.1 and 0.4 kg ha^-1^) than the other treatments. T3 reduced soil loss, runoff and nutrient losses to nearly a 100% while T1 and T2 reduced those losses from 43 to 88%. The losses of NO_3_-N and PO_4_-P were highly correlated with the amounts of runoff and eroded soil. Nutrient losses from the applied amendments were low (5.7% maximum), but the losses in the control could indicate long-term nutrient depletion in the soil (19 and 0.4 kg ha^-1^ of NO_3_-N and PO_4_-P, respectively). T1-T3 did not consistently increase crop yield or biomass in all three sites, but T1 increased both crop yield and biomass. We conclude that T3 (combining crop-residue mulch with organic amendment and runoff hedges) is the best treatment for steep slope areas but, the pigeon-pea hedges need to be managed for higher maize yield. T1 (combining organic amendment with soil surfactant) could be a better choice for flatter areas with deeper soils.

## Introduction

A combination of nutrient depletion, mismanagement of fragile ecosystems, and harsh climatic conditions can lead to soil degradation in arid and semiarid regions, particularly in the Sahel region, that threatens the sustainability of dryland agricultural systems [1–8]. Soil erosion, nutrient depletion, and other forms of land degradation reduce crop productivity per unit of water [9, 10] and affect water availability, quality, and storage. Water erosion and recurrent droughts, both important drivers of land degradation, limit crop productivity. Increasing water infiltration and storage in the soil is the key for increasing soil productivity in sub-Saharan Africa [11].

Erosion by water is the most common form of land degradation worldwide and usually increases with agricultural activity, particularly with annual cropping systems where the soil surface is seasonally exposed to rain with high intensities [4, 12]. Erosion removes nutrients, thins the soil layer, reduces rooting depth, damages soil structure, and reduces infiltration, resulting in negative nutrient balances and lower crop yields in most farming systems in West Africa [4, 5, 12, 13, 14] and parts of Asia [15–17].

Rainfed (or dryland) agriculture plays, and will continue to play, a dominant role in providing food and livelihoods for an increasing global population [8, 18]. Dryland agriculture produces most of the food consumed by poor communities in developing countries, with over 80% of the farmed land consisting of smallholder farms [19, 20]. Water-use efficiency, however, tends to be low [4, 9]. The productivity of dryland crops is particularly low in parts of sub-Saharan Africa and southern Asia, resulting in food insecurity and high levels of poverty in rural communities [10,21]. The depletion of nutrients from rainfed agricultural soils in many Asian, African, and Latin American countries is so high that current agricultural land use is not sustainable [4] and is considered the main biophysical factor limiting production on small-scale African farms [14, 16, 21]. The loss of nutrients and organic matter needed for plant growth are associated with surface erosion, because they are concentrated in the surface layer and are thus subject to loss with the eroded soil [5, 22]. Nutrient-use efficiency in cereal-based farming systems is also often very low due to shortages of moisture that limit the availability of nutrients [5,23], particularly in the Sahel region, where nutrient limitation is a major cause of the per capita decline in crop production [24].

Reversing land degradation is essential to improve water productivity, nutrient availability, and rural livelihoods in low-yielding dryland farming systems [4, 6]. Many measures of soil and water conservation (SWC), such as terraces, check dams, contour stone walls, contour ridges, afforestation, hedges, tillage, mulching, soil amendments, and water harvesting, have been widely used to improve soil quality, decrease erosion and nutrient losses, and increase infiltration and crop productivity [25–27].

Other factors that hinder crop productivity, however, must be simultaneously addressed to make use of the water gained from the mitigation of erosion-induced loss [10]. Nutrients and moisture are the primary factors limiting crop growth and productivity in sub-Saharan Africa [4, 28, 29], so maximising the use of rainwater without addressing nutrient deficiency is pointless. The development and adoption of sustainable systems of land management able to replenish or maintain soil nutrients and to control runoff and soil loss are thus crucial. The simultaneous improvement of soil fertility and water conservation promotes water-nutrient synergy [3, 10], and covering the soil with crop residues helps to reduce runoff [30–32]. The key factors for the sustainability of both short-term nutrient availability and long-term maintenance of soil organic matter under the systems of continuous dryland cultivation in sub-Saharan Africa may be the integration of water and nutrient management geared to ecologically sound and economically viable practices of land use [23]. The optimisation of dryland crop production requires a better understanding of the interaction between combined SWC measures and locally available nutrient sources (e.g. compost and animal or green manure).

Despite the positive impact of the widespread implementation of SWC measures to combat land degradation in Cabo Verde [33], the steep slopes and limited area of arable land, together with the semiarid and arid environments characterised by an irregular and poorly distributed rainy season with few heavy rains, continue to be challenges to dryland production. Maize (*Zea mays*) and beans, as the major dryland crops, occupy more than 80% of the dryland area under a low-input farming system that produces very low yields [34, 35]. Farmers in Cabo Verde, fearing economic losses due to the irregular rains, are reluctant to use fertilisers to improve the yield and soil fertility of their dryland fields. The only source of nutrients for the maize crop is the nitrogen (N) provided by intercropped beans. The application of fertilisers on the steep slopes, though, may pose an environmental risk due to flooding in the valleys [33].

Several studies have attempted to quantify soil erosion in Cabo Verde, both at plot and watershed levels [36–39], and some have evaluated techniques to reduce runoff and erosion [2, 30], but the quantification of the effects of these measures on nutrient losses associated with runoff and erosion, and their impact on soil nutrient balances, have been neglected. Furthermore, the findings of the few studies that have monitored crop productivity under different SWC measures in Cabo Verde [2, 40, 41] have been inconsistent.

This study evaluated the efficacy of selected strategies of soil-water management (residue mulching, a soil surfactant, and pigeon-pea hedges) combined with organic amendments (compost and animal and green manures) on N and P losses with eroded soil, and runoff and on crop yields in Cabo Verde drylands. We hypothesised that the synergetic effect of the combined strategies would significantly decrease nutrient losses related to runoff and erosion, and increase crop productivity.

## Materials and Methods

### 2.1. Study site

**Fig 1:**
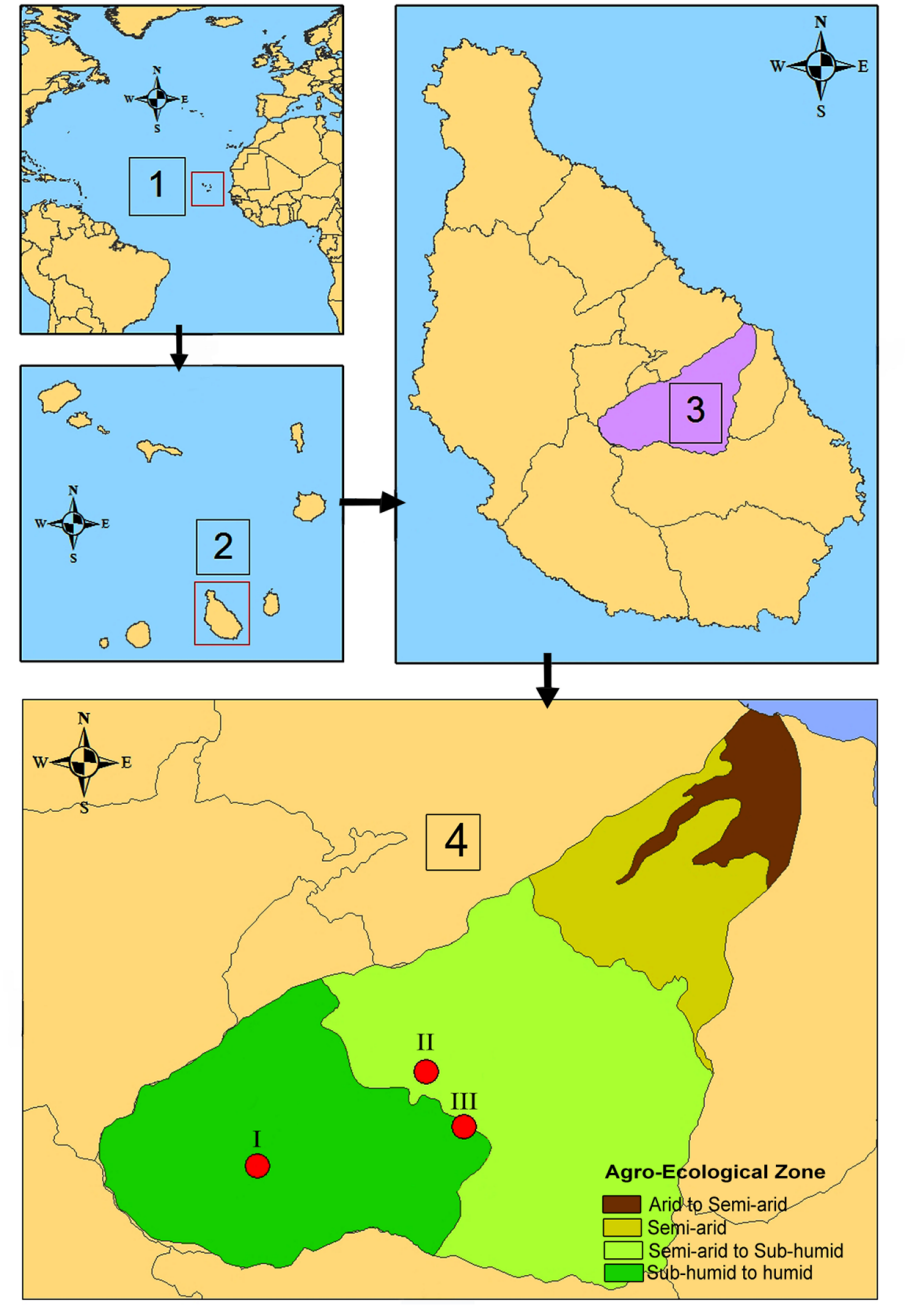
Location of (1) Cabo Verde, (2) Santiago, (3) the Ribeira Seca watershed, and (4) the experimental sites and Agro-Ecological zones. I, site I (São Jorge); II, site II (Serrado); and III, site III (Órgãos Pequenos). Adapted from [22]. Short title: Location of study sites within Ribeira Seca watershed, Santiago Island, and Cabo Verde.

This study was conducted at three sites: São Jorge, site I (1503'05.7''N, 2336'29.2''W); Serrado, site II (15°04'05.8'' N, 23°34'43.1''W); and Órgãos Pequenos, site III (15°03'25.3''N, 23°34'27.5''W) in the Ribeira Seca watershed (RSW), which is the largest (72 km²) watershed on Santiago, the main agricultural island of Cabo Verde (Fig 1). Site I was at the Instituto Nacional de Investigação e Desenvolvimento Agrário (INIDA) station and the president of the institute gave permission to conduct the study. Sites II and III were at private lands and the landowners gave permission to conduct the study. At none of the sites, the study did not involve endangered or protected species.

The climate is characterised by a monomodal rainfall regime, with a 3–4 month humid season (July-October) and an 8–9 month dry season (November-June). The mean annual rainfall is extremely heterogeneous and has an irregular spatiotemporal distribution, with annual precipitation varying from <200 mm downstream of the watershed to 650 mm upstream. The 30-year (1980–2010) mean annual rainfalls were 437, 300, and 310 mm at experimental sites I, II, and III, respectively, with most of the rain falling in August and September [42]. Rainfed (i.e. dryland) agriculture, comprising maize, several varieties of beans, and groundnuts, is the predominant land use, covering >83% of the area.

The sites were selected based on their specific characteristics of soil, agro-ecological zone (AEZ), slope, and agricultural practices. Site I is characterised by a low slope (<10%) and loamy Kastanozem soil and is on a terraced field at a research station in the subhumid to humid zone (351 m a.s.l. and mean annual rainfall of 437 mm). Site II is characterised by a steep slope (37%) and a sandy loamy Cambisol soil and is in the semiarid zone (183 m a.s.l. and mean annual rainfall of 300 mm). Site III is characterised by a moderately steep slope (23%) and a silt-clay-loam Regosol soil and is located on a farm at the junction of the semiarid and subhumid zones (204 m a.s.l and mean annual rainfall of 310 mm).

The initial physical and chemical properties of the soil (0–20 cm) varied among the three sites, but texture, bulk density, total N and extractable-P contents, and slope were homogeneous within each site (Table 1). The soils at sites I, II, and III had loam, sandy loam, and silt-clay-loam textures, respectively. Organic-matter content was low at sites II and III and average at site I. All sites were low in total N, particularly sites II and III with <10 mg N g^-1^ and the extractable-P content was average to high. Bulk density varied from 1.16 g cm^-3^ at site III to 1.42 g cm^-3^ at site I. The soil pHs were neutral to slightly alkaline. The rate of water infiltration was highest at site II and lowest at sites I and III.

**Table 1:** Soil properties (0–20 cm) at the experimental sites, site slopes, and total seasonal rainfall in 2012.

Site	Soil texture	Slope	pH (H_2_O)	Soil moisture	Bd*	OM**	Total N	P_ext_ ***	K****	Total rainfall
		(%)		(%)	(g cm^-3^)	(g kg^-1^)	(g kg^-1^)	(mg kg^-1^)	(mm h^-1^)	(mm)
I	Loam	8	7.3	7.2	1.42	20.6	13.7	13.5	17.86	572
II	Sandy loam	37	7.1	6.6	1.25	10.2	7.90	6.21	40.84	519
III	Silt-clay-loam	23	6.9	8.1	1.16	10.7	10.6	8.75	10.67	540

* Bd, bulk density;

** OM, organic matter;

*** extractable P;

**** K, infiltration rate.

### 2.2. Selection of technologies and treatments

The methodology used to assess and select the promising technologies combined collective learning and decision-making with the application of evaluated global best practices, which was a simplified form of the participatory approach developed by [43] and applied by [44].

The selection of the treatments followed the procedure described by [30]. Most of the potential techniques (e.g. crop-residue mulching, vegetation barriers, organic amendments, and improved planting pits) were selected from the database of the World Overview of Conservation Approaches and Technologies [45]. The local farmers who participated in the workshop for stakeholders, before the start of the field experiments, (1) identified the primary constraints of dryland production, (2) discussed potential technologies for addressing the constraints, (3) selected and ranked these technologies, and (4) grouped them into three categories representing low, medium, and high levels of investment.

The individual technologies for each study site were combined into three supplemented treatments (T1-T3), which were compared with an untreated control (T0). The supplemented treatments differed among the sites, depending on the local availability of residue mulch, the type of organic amendments, and the preferences of the local farmers [30]. Each supplemented treatment contained an organic amendment (compost or animal or green manure) and a water-management technique (residue mulch, a soil surfactant, and/or pigeon-pea (*Cajanus cajan*) hedges (Table 2).

**Table 2:** Descriptions of the treatments applied at each experimental site in 2012 (adapted from [22]) (fresh weights for compost with a moisture content of 35% and manure with a moisture content of 10%).

Treatment	Site I (São Jorge)	Site II (Serrado)	Site III (Ó. Pequenos)
T0 (Control)	Traditional maize/bean intercropping (no input)	Traditional maize/bean intercropping (no input)	Traditional maize/bean intercropping (no input)
T1	Animal manure (4 t ha^-1^) + soil surfactant (1 mL m^-2^)	Compost (4 t ha^-1^) + soil surfactant (1 mL m^-2^)	Animal manure (4 t ha^-1^) + soil surfactant (1 mL m^-2^)
T2	Compost (4 t ha^-1^) + soil surfactant (1 mL m^-2^)	Pigeon-pea hedges + animal manure (4 t ha^-1^) + soil surfactant (1 mL m^-2^)	Pigeon-pea hedges + green manure (1 t ha^-1^ *Leucaena leucocephala* prunings) + soil surfactant (1 mL m^-2^)
T3	Mulch (4 t ha^-1^ banana leaves) + compost (4 t ha^-1^)	Mulch (4 t ha^-1^ *Panicum maximum* grass) + pigeon-pea hedges + animal manure (4 t ha^-1^)	Mulch (4 t ha^-1^ *P*. *maximum* grass) + pigeon-pea hedges + green manure (1 t ha^-1^ *L*. *leucocephala* prunings)

The compost used in the experiments was prepared four months before the rainy season from raw organic materials (cow and poultry manure, ash, various dry crop residues, and *Leucaena leucocephala* prunings) enriched with 3 kg of a nitrogen/phosphorus/potassium (NPK) inorganic fertiliser. All materials were added in layers to fill a 1-m deep 2 × 1 m rectangular pit at the INIDA research station in Cabo Verde, watered weekly and then biweekly in a second pit for three months. The decomposed material was then covered and left to rest until its application in the field. The animal manure used at all sites was obtained from a farmyard and consisted of a well-decomposed mixture of cow manure and animal feed (i.e. crop residue). The green manure consisted of chopped fresh twigs of *L*. *leucocephala* obtained from nearby shrubs planted on the steep slopes for conserving soil and water.

Table 3 presents the nutrient contents of the organic amendments applied in the two study seasons. Total N, P, and K contents of the organic amendments were analysed using STH soil-test kits (LaMotte, Eijkelkamp, NL): N and P by the colorimetric method.

**Table 3:** Nutrient contents (total N, P, K) (g kg^-1^) of the organic amendments (dry weights) used in the 2012 cropping season.

Amendment	N	P	K
Animal Manure	24.2	3.00	16.2
Compost	23.2	2.30	24.1
*Leucaena* green manure	38.1	2.20	17.0

### 2.3. Experimental set-up

**Fig 2:**
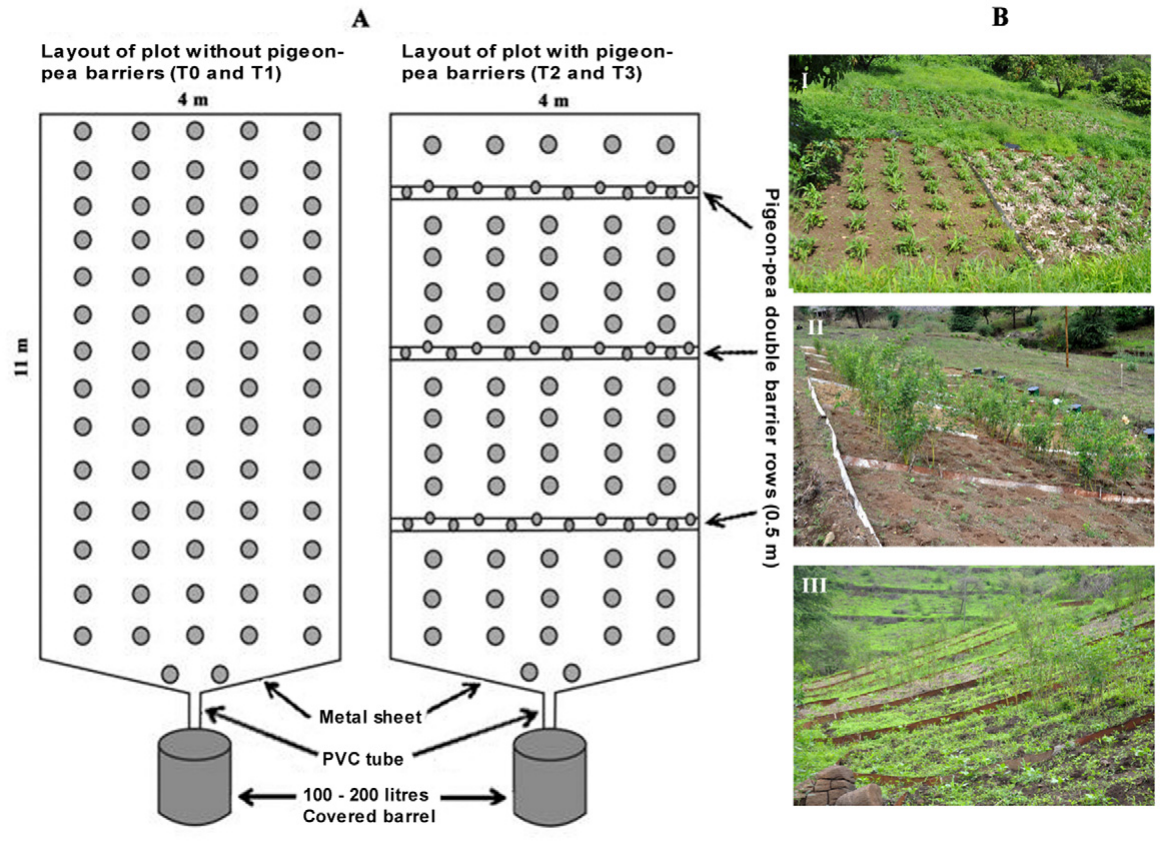
Layout and aspects of the experimental plots. (A) Schematic layout of the 11 × 4 m erosion plots (sites II and III) with and without hedges, and (B) field layouts of the plots at sites I, II, and III. Short title: Layout and aspects of the experimental plots.

The experiments were conducted during the 2012 rainy season, from August to October. The experimental plots were 11 × 4 m in the two farm trials (Fig 2) and 6 × 4 m in the trial at the research station. Each experimental plot was isolated with a metal sheet of 20 cm depth to prevent the entrance of runoff water from the other plots as well from subsurface flow. Despite different plot sizes, a pre-study indicated similar erosion and infiltration depth for the different sites. A barrel was placed at the bottom of each plot to collect runoff water. The experiments had a randomised design with three replicates of three supplemented treatments and one control. Fig 2A and 2B shows the field layout of the plots at each study site.

For T1-T3, planting pits (20 cm wide and 15 cm deep) were dug with a hoe 75 cm apart in the rows, with 80 cm between the rows. The organic amendments (compost or animal or green manure) were applied manually to the bottoms of the pits and covered with a small amount of soil before seeding. The agricultural soil surfactant IrrigAid Gold ACA 1848 (Aquatrols, New Jersey, USA) was diluted in water and sprayed on the soil surface with a hand-pressure atomiser after seeding. Three types of crop residue (banana leaves, sugarcane leaves, and P. maximum grass) were cut into small pieces and applied to the surface as mulch (4 t ha^-1^) to cover 60–80% of the soil surface.

### 2.4. Crop management and measurements of crop growth and yield

The crops used in the experiments were maize and two local types of beans (*Vigna unguiculata*, or cowpea, and *Lablab purpureus*, or feijão pedra). The crops were planted after the first significant rain (>20 mm) by placing three maize and four bean seeds (two of each type of bean) in each planting pit, thus forming a seed cluster. For the plots with pigeon-pea hedges (T2-T3), two pigeon-pea seeds were alternated along two lines 50 cm apart to form double-row hedges 3 m apart (Fig 2A). The maize and beans were planted between the hedges. The planting density was approximately 16 300 seed clusters per hectare in all plots.

Weeds were removed from all plots twice during the rainy season, approximately three and six weeks after planting (Table 4), either with a hoe or by hand, with minimum disturbance of the soil, except for the control plots where weeding was always with a hoe. The heights of the maize plants were measured in all plots with a measuring tape, from the soil surface to the tip of the first fully developed leaf, taking six measurements per plot at flowering.

**Table 4:** Dates of planting, weeding, and harvesting at each experimental site for the 2012 cropping season.

Field operation	Site I	Site II	Site III
1^st^ significant rain	Aug 24	Aug 24	Aug 24
Sowing of maize and beans	Aug 27	Aug 28	Aug 28
1^st^ weeding	Sep 17	Sep 19	Sep 20
Harvest of *V*. *unguiculata* (cowpea)	Nov 08	Nov 09	Nov 07
Harvest of maize	Dec 19	Dec 20	Dec18
Harvest of *L*. *purpureus* (feijão pedra)	Mar 20 2013	Mar 18 2013	Mar 25 2013

Composite samples of maize leaves were collected in each plot at the early maturity stage for the analysis of nutrient content at BLGG AgroXpertus, Wageningen, NL, using near-infrared spectroscopy for N and SPZ2 for P and K (www.blgg.com).

The maize and beans, excluding the outer rows, were harvested when dry. The yield of the dry maize was determined by weighing all cobs harvested from each plot. The bean yield was determined by weighing the dry grains harvested from each plot. Crop biomass, which consisted of all maize and bean plant material, was cut at the soil surface and weighed for all plots.

### 2.5. Data collection and calculations

Three composite soil samples were collected to a depth of 20 cm from each site at the end of the dry season before the experiments began. Laboratory analyses were conducted at INIDA. Soil texture was determined by the pipette method, bulk density by the core method [46], infiltration rate by minidisk infiltrometry (Decagon, Pullman, WA, USA), pH (H2O) by a potentiometer, N content by Kjeldahl digestion [47], extractable-P by the Olsen method [48], and organic-matter content by the Walkley Black method [49]. Daily rainfall was measured with a simple rain gauge installed at each experimental site.

Automatic Em5b data loggers with moisture sensors (Decagon Devices, Pullman, WA, USA) were installed in each plot at a depth of 15 cm (only one at each site due to financial restrictions) to register trend in the volumetric moisture content of the soil every 30 min under the various treatments. Daily moisture contents were estimated by averaging the 30-min values. Runoff water and eroded soil were collected after each erosive rain following the procedure described in [30]. After measuring the volume of the runoff, 1 L was taken to the laboratory for analysis. NO_3_ concentrations were determined by the Aquamerck colorimetric method, PO_4_ concentrations were determined by Olsen spectrophotometry [48], and the pH was measured potentiometrically.

After filtration and oven-drying, the eroded soil was weighed, and the amount of soil loss (g m^-2^) was estimated for each plot. The seasonal soil loss per plot was calculated by totalling the amount of soil lost from each erosive rain. The seasonal runoff coefficient (Cr) was calculated for total growing-season erosive periods of rain using the equation [30]:

Cr=(Q/R)×100

where Q is the runoff volume (in mm) and R is the total rainfall producing runoff (in mm). The runoff and eroded soil were considered to be the only sources of nutrient losses (see [30] for detailed data analyses of the runoff and sediments).

NO_3_ and PO_4_ concentrations in the runoff and eroded soil were determined by colorimetry using STH soil-test kits (LaMotte, Eijkelkamp, NL). Nutrient losses (NO_3_-N and PO_4_-P) per plot and from each rain in the runoff and sediments were estimated by [22]:

$$\text{nutrient loss in runoff }\left(\text{mg}\cdot {\text{m}}^{-2}\right)=\text{nutrient concentration in runoff }\left(\text{mg}\cdot {\text{L}}^{-1}\right)\times \text{total runoff }\left(\text{L}\right)$$ (1)

$$\text{nutrient loss in eroded soil }\left(\text{mg}\cdot {\text{m}}^{-2}\right)\text{nutrient concentration in eroded soil }\left(\text{mg}\cdot {\text{g}}^{-1}\right)\times \text{total soil loss }\left(\text{g}\cdot {\text{m}}^{-2}\right)$$ (2)

Partial NO_3_-N and PO_4_-P balances were estimated from the amounts added with the organic amendments and the amounts lost in the runoff and sediments.

### 2.6. Data Analysis

All statistical analyses were performed using IBM SPSS 19.0 statistics software. The data were tested for normality with the Kolmogorov-Smirnov test. Analyses of variance tested for the significance of the supplemented treatments on plant height, crop and biomass yields, seasonal NO_3_-N and PO_4_-P losses in the runoff and eroded soil. We used the post-hoc Dunnett’s T3 test for non-homogenous variances to identify significant differences among the treatments. All tests were deemed statistically significant at a probability value of 0.05. A principal component analysis (PCA) was conducted for treatment, seasonal runoff and eroded soil, site slope, seasonal rainfall, seasonal NO_3_-N and PO_4_-P losses, soil moisture, yield, and biomass. Components with eigenvalues over Keiser’s criterion of 1 were extracted. Pearson correlation analyses of the variables were also performed.

## Results

### 3.1. Rainfall distribution

**Fig 3:**
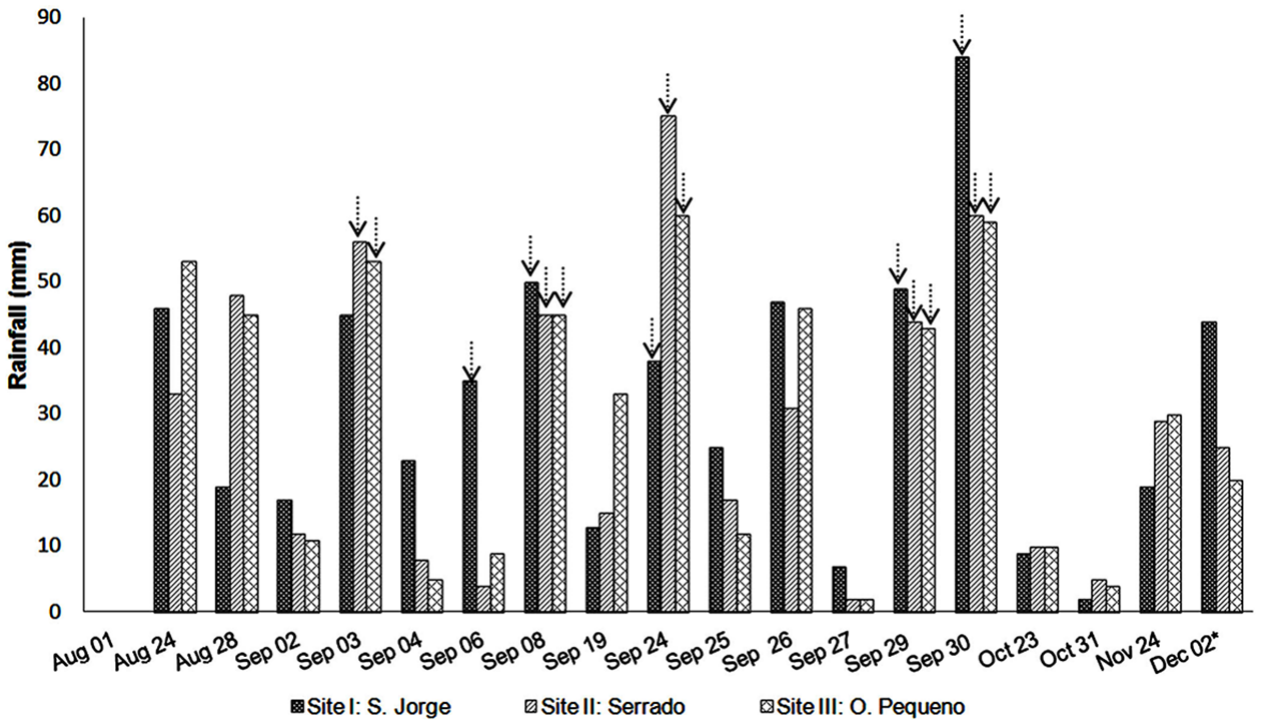
Rainfall distribution over the 2012 rainy season at the three experimental sites. The arrows indicate daily rainfall causing runoff. * Rainfall accumulated between Nov 30 and Dec 2. Short title: Rainfall distribution over the 2012 rainy season.

The total seasonal rainfalls in the 2012 rainy season were 572, 519, and 540 mm at sites I, II, and III, respectively (Table 1), which were substantially higher than the average precipitation for August to October for 1980–2010. The seasonal rainfall did not substantially differ among the sites, but the distribution varied considerably (Fig 3). The first rain fell in late August, and rains were well distributed throughout September, with 12 days of rain, but no rain fell in October. Of 18 total rainy days, five rainfalls were <20 mm, only one was >50 mm, and the others were 20–50 mm for all three sites (Fig 3).

### 3.2. Effects of the treatments on soil moisture

**Fig 4:**
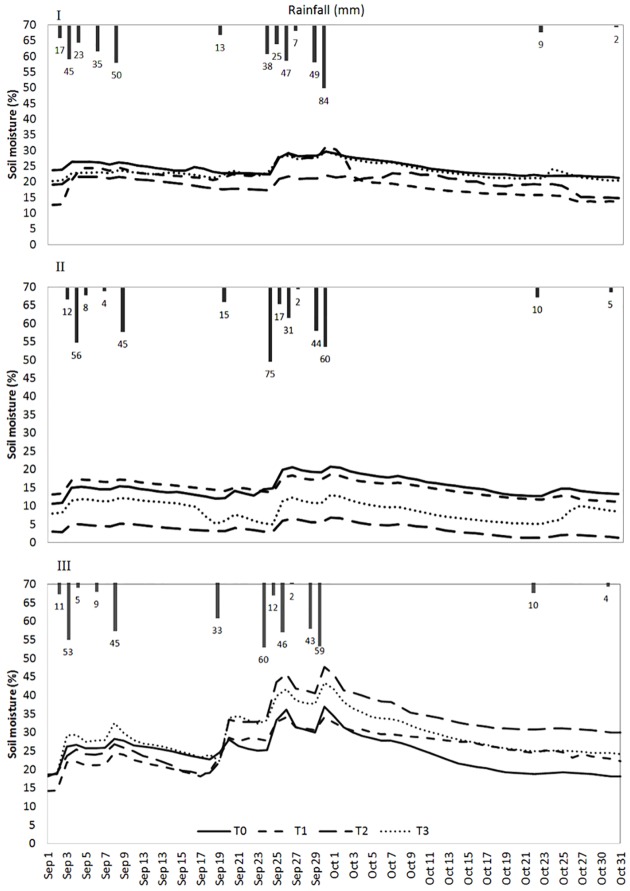
Daily soil-moisture trends (% by volume) in the planting pits of the various treatments during the 2012 rainy season. The bars represent rainfall per rain. The higher moisture peaks correspond to the rains. Short title: Daily soil-moisture trends in the planting pits.

Continuous soil-moisture trends at a depth of 15 cm in the planting pits exhibited different behaviours among the three sites and throughout the growing season (Fig 4).

At site I, the moisture contents of the supplemented treatments were either identical to or lower than the control, except during the wet period after the heavy rain of Sep 29 when the moisture content was higher in T1 (Fig 4I). The moisture content in T1, however, was lower than the contents of the other treatments during dry periods. T3 had similar moisture contents as T0 throughout the season, while the other supplemented treatments had lower moisture contents. The moisture contents in the treatments had the order T0≥T3>T1>T2.

At site II, T1-T3 had soil-moisture contents lower than that in T0, except for the first month of the season when T2 had a higher moisture content (Fig 4II). The moisture contents had the order T0≥T1>T3>T2. T2 had a very low moisture content throughout the experimental season, and the content did not vary a lot following rains.

At site III, the supplemented treatments contributed to increases in soil-moisture content in the planting pits relative to the control (Fig 4III) and the increases were larger after long periods of drought. Early in the season when plant cover was low, soil moisture was higher than in T0 only in T3.

The soil-moisture contents of the planting pits differed among the sites, being highest (14–48%) at site III, lowest (3–21%) at site II, and intermediate (13–30%) at site I.

### 3.3. Effects of the treatments on nutrient losses in the runoff and sediments

#### 3.3.1. Runoff and soil loss

All supplemented treatments produced significantly less seasonal runoff than the control, with T3 significantly outperforming T1 and T2 at all three sites (Table 5). T3 had the lowest amount of seasonal runoff (0.1 L m^-2^) at site I, and T0 had the highest amount (20 L m^-2^) at site II. T3 reduced seasonal runoff relative to T0 by 95% (site III) to 99% (sites I and II). Runoff generation was in the orders T3<T1 = T2<T0 at site I and T3<T1 = T2 = T0 at sites II and III, indicating significant differences.

**Table 5:** Effects of the treatments on seasonal runoff, runoff coefficient, seasonal soil loss and concentrations of nutrients (mean ± standard deviation) in the runoff and eroded soil. Different letters indicate significant differences (Dunnett’s T3 test) between the treatments within the same site (*P*<0.05): a<b<c. See Table 2 for treatment descriptions.

Site	Treatment	Runoff (L m^-2^)	Runoff coefficient (%)	Eroded soil* (g m^-2^)	NO_3_-N in runoff (mg L^-1^)	PO_4_-P in runoff (mg L^-1^)	NO_3_-N in eroded soil (mg g^-1^)	PO_4_-P in Eroded soil (mg g^-1^)
I	T0	12.1±1.76c	4.1±0.5	38.9±17.6c	3.24±2.71a	0.06±0.06a	5.30±3.61a	18.8±13.7a
	T1	6.81±1.08b	2.3±0.3	8.99±3.66b	2.20±1.67a	0.06±0.07a	4.37±3.03a	14.7±12.6a
	T2	8.33±2.65b	3.0±0.8	9.90±6.42b	2.51±1.99a	0.03±0.03a	3.45±2.25a	12.8±10.2a
	T3	0.11±0.19a	0±0.1	0.02±0.01a	2.08±2.03a	0.04±0.06a	2.44±1.71a	8.71±6.81a
II	T0	20.1±0.23c	6.7±0.1	104±18.4c	4.40±2.26a	0.07±0.05a	2.80±1.65a	8.30±4.15a
	T1	17.6±0.53bc	5.7±0.2	35.2±16.4b	3.06±2.12a	0.02±0.01a	2.24±1.21a	5.78±4.53a
	T2	15.4±2.29b	5.1±0.7	48.1±18.4b	3.24±2.03a	0.04±0.03a	1.30±1.14a	5.27±3.56a
	T3	0.17±0.10a	0.1±0.0	0.05±0.04a	3.85±2.18a	0.06±0.04a	2.38±1.90a	5.62±3.18a
III	T0	16.4±1.64b	5.6±0.6	156±15.6c	4.83±2.64a	0.06±0.04a	8.51±3.97a	17.8±8.88a
	T1	14.8±1.59b	5.1±0.5	41.0±15.1b	2.51±1.71a	0.03±0.05a	5.92±3.46a	13.3±8.25a
	T2	11.3±4.25b	3.9±1.4	25.0±14.4b	2.69±1.70a	0.04±0.02a	6.29±4.21a	12.8±7.04a
	T3	0.75±1.30a	0.3±0.1	0.68±0.12a	3.85±2.18a	0.03±0.02a	6.72±3.37a	13.2±6.12a

*mg m^-2^ = 10^−2^ kg ha^-1^.

Similarly, all supplemented treatments generally lost significantly (*P*<0.05) less soil than the control, with T3 registering the lowest amount of soil loss at all three sites (Table 5). Site III lost the most soil, reaching 1.6, 0.4, 0.3, and 0.01 Mg ha^-1^ in T0, T1, T2, and T3, respectively. T1 did not generally differ significantly (*P*>0.05) from T2, but both lost significantly (*P*<0.05) less soil than T0. The order of soil-loss reduction was T3<T1 = T2<T0 for all sites (see [30] for details).

#### 3.3.2. NO_3_-N and PO_4_-P concentrations in runoff and eroded soil

Nutrient (NO_3_-N and PO_4_-P) concentrations in the runoff and eroded soil did not differ significantly among the supplemented treatments at any of the sites relative to T0 (Table 5). The NO_3_-N concentration (2.20–4.83 mg L^-1^), however, was higher than the PO_4_-P concentration (0.02–0.07 mg L^-1^) in the runoff, and the PO_4_-P concentration (5.27–18.8 mg g^-1^) was higher than the NO_3_-N concentration (1.30–8.51 mg g^-1^) in the eroded soil. Nutrient concentrations in the runoff from the supplemented treatments were very similar among the sites but differed from those in the eroded soil in the orders III>I>II for NO_3_-N and I≥III>II for PO_4_-P. The NO_3_-N concentrations in runoff ranged from 2.20 mg L^-1^ (T1, site I) to 4.83 mg L^-1^ (T0, site III), and those of PO_4_-P ranged from 0.02 mg L^-1^ (T1) to 0.07 mg L^-1^ (T0) both at site II. In the eroded soil, the NO_3_-N concentrations ranged from 1.30 mg g^-1^ (T2, site II) to 8.51 mg g^-1^ (T0, site III) and those of PO_4_-P ranged from 5.27 mg g^-1^ (T2, site II) to 18.8 mg g^-1^ (T0, site I). The mean NO_3_-N concentrations in the runoffs were 2.51, 3.64, and 3.47 mg L-1 for sites I, II, and III, respectively, and the mean PO_4_-P concentration was 0.05 mg L-1 for all sites. The mean NO_3_-N concentrations in the eroded soil were 3.89, 2.20, and 6.86 mg g-1, and the mean PO_4_-P concentrations were 13.8, 6.18, and 14.3 mg g-1, at sites I, II, and III, respectively (Table 5).

#### 3.3.3. NO_3_-N and PO_4_-P losses

**Fig 5:**
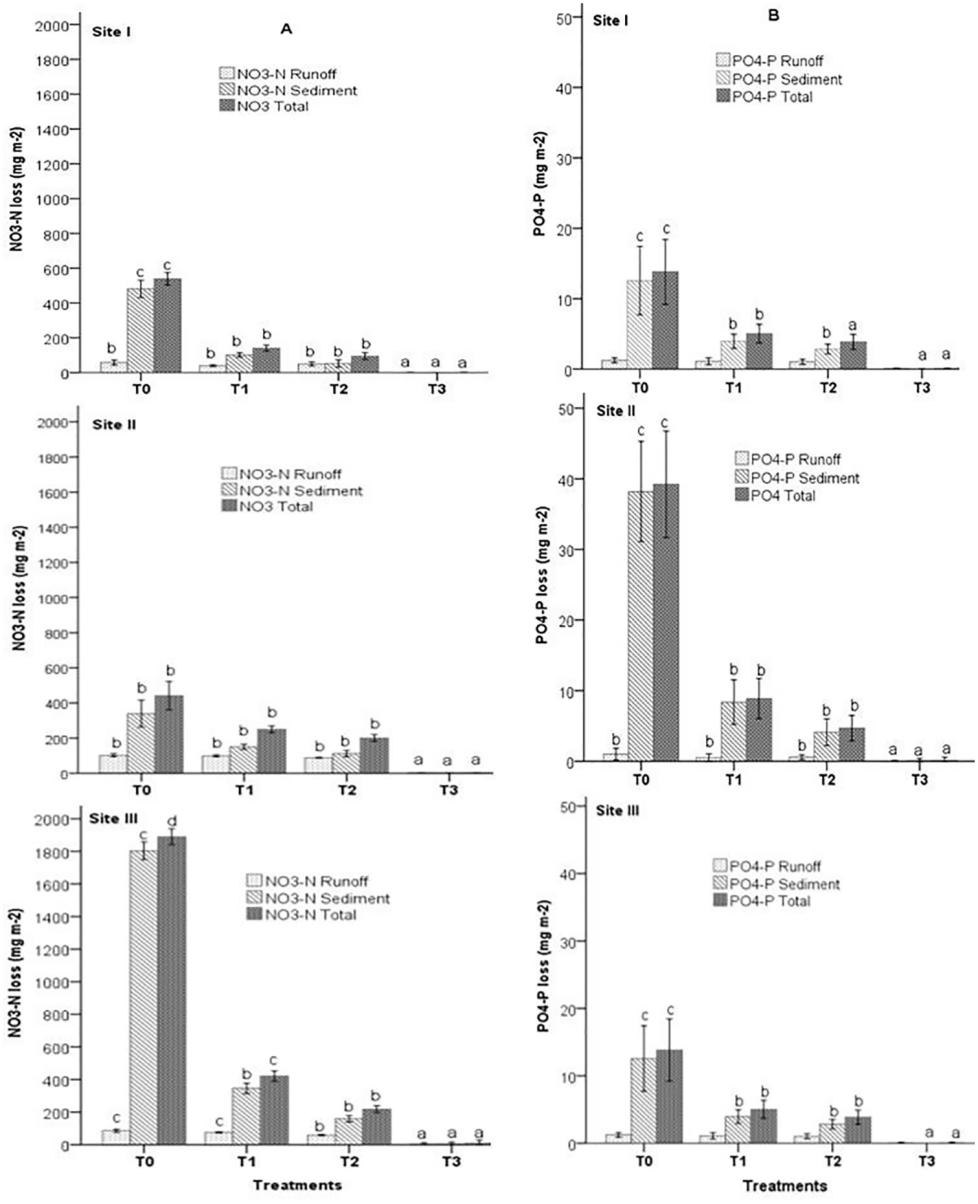
Annual losses of (A) NO_3_-N and (B) PO_4_-P in the eroded soil and runoff for the various treatments. Different letters indicate significant differences (Dunnett’s T3 test) within the same site and category (P<0.05): a<b<c<d. Error bars are standard deviation. See Table 2 for treatment descriptions. Short title: Annual losses of NO_3_-N and PO_4_-P in the eroded soil and runoff.

The amounts of runoff and soil loss in 2012 were low, but the treatments had highly significant effects on the losses of both NO_3_-N and PO_4_-P related to runoff and eroded soil (Fig 5) at all sites. T0 had the significant highest (*P*<0.01) and T3 the significant lowest (P<0.01) seasonal NO_3_-N loss at all sites (Fig 5A). T0 had losses of 539, 442, and 1890 mg NO_3_-N m^-2^ at sites I, II, and III, respectively, with 89, 77, and 95% lost with the eroded soil and 11, 23 and 5% lost in the runoff (Table 6). Both T1 and T2 at site I lost significantly (*P*<0.05) less NO_3_-N than T0, but the losses did not differ between them. At site III, T3 lost significantly less total NO_3_-N than T1 and T2.

**Table 6:** Contributions of runoff and eroded soil to seasonal NO_3_-N and PO_4_-P at each experimental site in the various treatments. See Table 2 for treatment descriptions.

Site	Treatment	NO_3_-N loss (%)		PO_4_-P loss (%)	
		Runoff	Eroded soil	Runoff	Eroded soil
I	T0	11	89	8	92
	T1	28	72	13	87
	T2	49	51	28	72
	T3	80	20	0	100
II	T0	23	77	9	91
	T1	40	60	21	79
	T2	44	56	27	73
	T3	89	11	100	0
III	T0	5	95	3	97
	T1	18	82	6	94
	T2	27	73	12	88
	T3	44	56	27	73

NO_3_-N at sites I and II was lost mainly with the eroded soil in T0-T2 and mainly in the runoff in T3. N was lost at site III mainly with the eroded soil in all treatments. Total NO_3_-N losses in T3 were negligible at sites I and II and low (9.36 mg m^-2^) at site III. The losses in T1 and T2 were not significantly different.

Similarly, to NO_3_-N, total PO_4_-P loss was significantly highest in T0 and significantly lowest in T3 at all sites (Fig 5B). T0 had losses of 16.6, 13.8, and 39.2 mg PO_4_-P m^-2^ at sites I, II, and III, respectively (Fig 5B), with >90% in the sediments and <10% in the runoff (Table 6). Both T1 and T2 lost significantly (*P*<0.05) less PO_4_-P than T0 at sites I and III, but the losses did not differ between them. At site II, T3 lost significantly less total PO_4_-P than T1 and T2. The amount of PO_4_-P lost in the sediments was higher for T1 than for T2.

The runoff and soil losses, NO_3_-N, and PO_4_-P were low, but all supplemented treatments substantially reduced the losses relative to the control (Table 7). T3 reduced the loss of all parameters by nearly 100% at all sites. The reductions in the losses of total NO_3_-N varied from 43 to 78% in T1 and from 55 to 88% in T2. Similar values and trends were observed for PO_4_-P. The reductions in the losses of all parameters were lowest at site II and highest at site III.

**Table 7:** Effects of the treatments on the seasonal reduction of runoff, soil loss, NO_3_-N and PO_4_-P losses relative to the control at each experimental site (%). See Table 2 for treatment descriptions.

Site	Treatment	Runoff (%)	Eroded soil (%)	NO_3_-N (%)	PO_4_-P (%)
I	T0	0	0	0	0
	T1	44	77	74	76
	T2	31	75	83	83
	T3	99	100	100	100
II	T0	0	0	0	0
	T1	12	66	43	64
	T2	23	54	55	72
	T3	99	100	100	100
III	T0	0	0	0	0
	T1	10	74	78	77
	T2	31	84	88	88
	T3	95	100	100	100

#### 3.3.4. Nutrient inputs versus losses

The total nutrients (N and P) added with the organic amendments in T1-T3 were higher than the nutrient losses as NO_3_-N and PO_4_-P in the runoff and eroded soil (Table 8). The fractions of the nutrient inputs lost in the runoff and sediments were very low in all treatments, with the NO_3_-N losses ranging from negligible in T3 at all sites to 5.7% from the N added by the amendments in T2 at site III. T0 had the highest nutrient losses at all sites and T3 had the lowest. T0, which received no nutrient inputs, lost 5.39 and 0.17 kg ha^-1^ of NO_3_-N and PO_4_-P, respectively, at site I and 4.42 and 0.14 kg ha^-1^, respectively, at site II. The losses were higher at site III, with 18.9 and 0.4 kg ha^-1^ for NO_3_-N and PO_4_-P, respectively. The losses of both nutrients were negligible in T3 at all sites, indicating the effectiveness of this treatment in reducing nutrient losses.

**Table 8:** Total N and P input from the organic amendments, their losses as NO_3_-N and PO_4_-P in the runoff and eroded soil for the various treatments at each experimental site in the 2012 cropping season. Different letters indicate significant differences (Dunnett’s T3 test) within the same site (*P*<0.05): a<b<c<d. See Table 2 for treatment descriptions.

Site	Treatment	Inputs from amendments		Losses in runoff and sediments		Losses from input nutrients	
		Total N (kg ha^-1^)	Total P (kg ha^-1^)	NO_3_-N (kg ha^-1^)	PO_4_-P (kg ha^-1^)	NO_3_-N (%)	PO_4_-P (%)
I	T0	0	0	5.39c	0.17c		
	T1	87.9	10.9	1.40b	0.04b	1.60	0.37
	T2	60.3	6.0	1.01b	0.03b	1.70	0.50
	T3	60.3	6.0	0.01a	0a	0.02	0
II	T0	0	0	4.42b	0.14c		
	T1	60.3	6.0	2.5b	0.05b	4.10	0.83
	T2	87.9	10.9	2.0b	0.04b	2.20	0.37
	T3	87.9	10.9	0.01a	0a	0.01	0
III	T0	0	0	18.9d	0.4c		
	T1	87.9	10.9	4.21c	0.09b	4.80	0.83
	T2	38.1	2.22	2.18b	0.05b	5.70	2.30
	T3	38.1	2.22	0.1a	0a	0.27	0

### 3.4. Effects of the treatments on crop growth and yield

#### 3.4.1. Crop growth and yield

Maize plant height differed significantly (*P*<0.05) among the treatments only at site II, in the order T1≥T3>T0 = T2 (Table 9). Maize development varied among the sites, with plant height following the order I>III>II. Except at site III, the treatments significantly (*P*<0.05) affected maize yield, but the effect was different at each site (Table 9). At site I, T1 and T3 (1.4 and 1.3 t ha^-1^, respectively) significantly (*P*<0.05) increased maize yield relative to T0 (0.6 ton ha^-1^), with no significant difference between the two or between T2 and T0. Maize yield followed the order T3≥T1>T2 = T0. At site II, only T1 (0.8 ton ha^-1^) significantly increased maize yield relative to T0 (0.6 ton ha^-1^). Maize yield had the order T1>T0 = T3 = T2. T1-T3 did not differ significantly from T0 at site III. Mean maize yield was low at all sites in the order I>II>III (Table 9).

**Table 9:** Effects of the treatments on maize height, maize yield, bean yield, and crop biomass (dry biomass) at each experimental site for the 2012 growing season. The values are means ± standard deviations. Different letters indicate significant differences between the treatments (*P*<0.05): a<b<c. See Table 2 for treatment descriptions.

Site	Treatment	Maize height (cm)	Maize yield (kg ha^-1^)	Bean yield* (kg ha^-1^)	Biomass** (kg ha^-1^)
I	T0	172±7.0	625±110a	88.3±11a	4722±636a
	T1	202±9.0	1306±127b	174±52b	6736±1147b
	T2	191±8.0	930±122a	81.7±10a	6111±636b
	T3	205±16	1430±307b	89.9±13a	7778±1339b
II	T0	148±12a	549±144a	458±155b	3409±601a
	T1	171±7.0b	761±156b	492±165b	4394±860b
	T2	151±10a	377±55a	188±9.46a	2719±402a
	T3	168±10b	478±122a	230±49.8a	3070±402a
III	T0	163±12	258±57	437±205	2972±88a
	T1	171±2.0	320±47	530±159	3523±227b
	T2	163±5.0	241±44	249±22.9	2193±76a
	T3	171±9.0	236±34	326±85.8	2500±132a

* Bean yield is for both feijão pedra and cowpea, except at site I where it is for cowpea only;

**Biomass includes all aboveground material from the maize, feijão pedra, and cowpea plants after harvesting.

The treatments significantly affected bean yield at sites I and II but not at site III (Table 9). At site I, T1 significantly increased cowpea yield relative to T2, T3, and T0, nearly doubling the yield. The bean yields at site II were significantly lower (50% or less) in T2 and T3 than in T0, while the yield in T1 was similar to that in T0.

The treatments significantly affected crop biomass at all sites in the 2012 cropping season (Table 9). All supplemented treatments at site I significantly increased biomass relative to T0, but only T1 significantly increased biomass at sites II and III.

#### 3.4.2. Nutrient status of the maize plants

The application of all three types of organic amendments (compost and animal and green manure) increased the N contents of the maize leaves relative to the control, but the increases were not statistically significant (Table 10). The N content varied from 16.9 to 22.5 g kg^-1^, and the P content varied from 3.36 to 4.30 g kg^-1^.

**Table 10:** Nitrogen (N) and phosphorus (P) content of maize leaves at each experimental site for the 2012 cropping season. Different letters indicate significant differences between the treatments (*P*<0.05): a<b<c. The leaf samples were collected at early maturity.

Treatment*	Site I		Site II		Site III	
	N**	P**	N**	P**	N**	P**
	(g kg^-1^)	(g kg^-1^)	(g kg^-1^)	(g kg^-1^)	(g kg^-1^)	(g kg^-1^)
T0	17.7±2.55	4.27±0.97	18.4±1.42	2.75±0.134a	16.8±3.67	3.20±0.60
T1	20.6±3.79	4.34±0.39	20.0±1.89	3.36±0.5ab	18.8±2.81	3.37±0.77
T2	22.5±4.24	4.63±0.76	21.7±3.12	4.30±0.55bc	20.5±2.22	3.43±1.24
T3	19.6±1.71	4.37±0.31	21.5±2.25	5.13±0.06c	21.7±2.45	2.97±0.86

* See Table 2 for treatment descriptions.

**Sufficiency range is 25–35 g kg^-1^ for N and 2.5–4.0 g kg^-1^ for P [40].

### 3.5. Relationships between the various parameters

**Fig 6:**
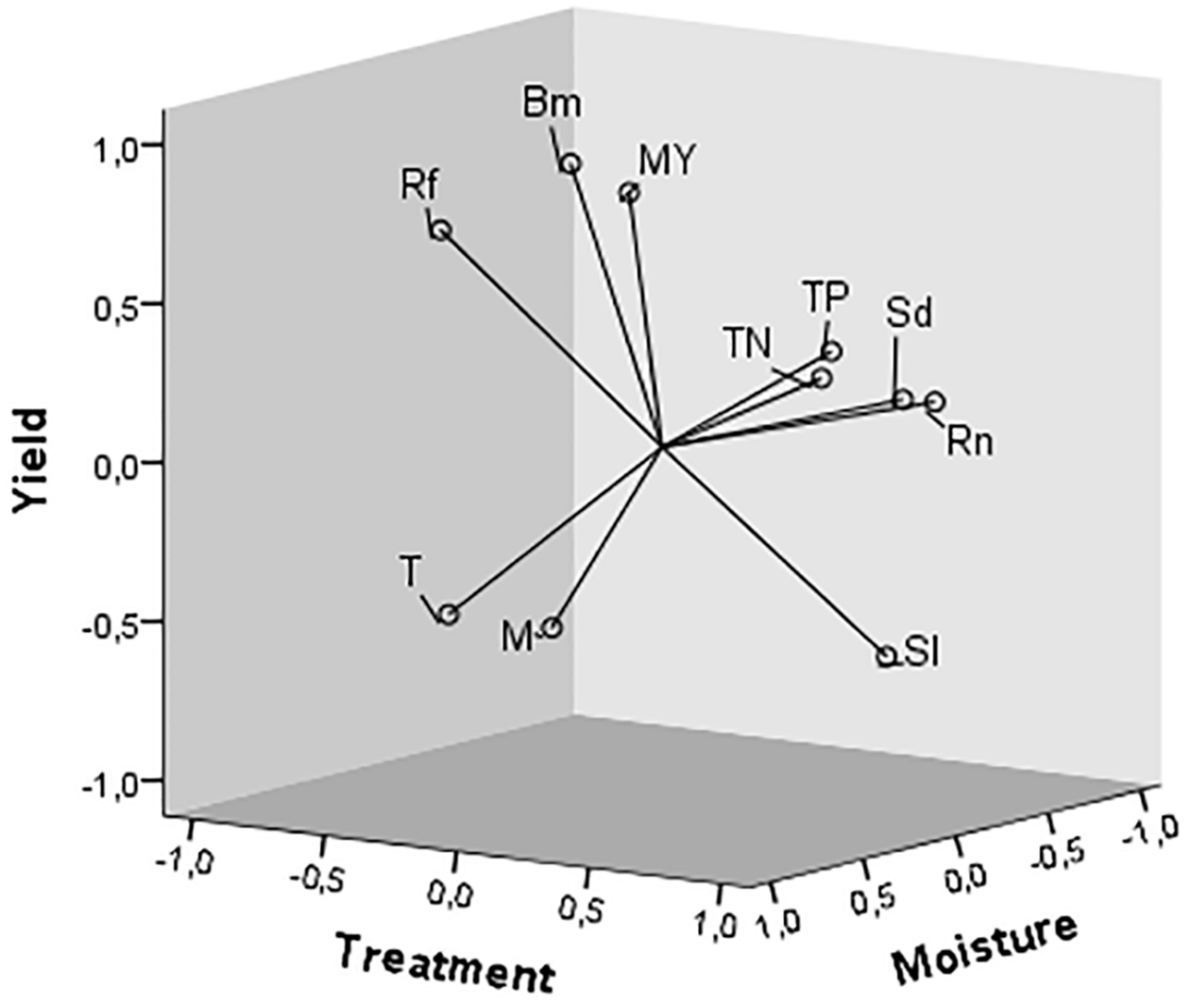
Principal components showing the three components extracted from the variables: T, treatment; Rf, rainfall; Bm, biomass; MY, maize yield; TN, total N loss; TP, total P loss; Rn, runoff; Sd, sediment loss; Sl, slope; M, soil moisture. Short title: Principal components.

Three principal components (PCs) together explained 89% of the variance in the data. Parameters such as runoff and soil loss, NO_3_-N, and PO_4_-P loss were associated with PC1 (treatment component), which explained 43% of the variance, while maize yield, crop biomass, rainfall, and slope were associated with PC2 (yield/crop component), which explained 30% of the variance. Only soil moisture was associated with PC3, which explained 16% of the variance (Fig 6 and Table 11).

**Table 11:** Component matrix of the relationships between the parameters and the main components of the principal components analysis.

Parameter	Component		
	1	2	3
Treatment	-0.707	-0.577	0.143
Biomass	-0.458	0.822	-0.160
Maize yield	-0.400	0.703	-0.393
Runoff	0.792	0.172	-0.344
Soil loss	0.928	0.247	0.024
Soil moisture	0.135	-0.441	0.788
Rainfall	-0.517	0.697	0.457
Total N loss	0.832	0.348	0.327
Total P loss	0.834	0.426	0.276
Slope	0.499	-0.681	-0.502

Runoff and soil loss were strongly positively correlated (α = 0.001) at all sites, with the order of correlation of I (0.800)>II (0.790)>III (0.693). The losses of NO_3_-N and PO_4_-P were strongly positively correlated with the amount of soil lost, which was also highly positively correlated with the runoff. Maize yield and biomass were positively correlated with soil moisture, rainfall, and slope but were not significantly correlated with treatment application or the nutrient parameters.

Both runoff and soil loss were positively correlated with rainfall, total NO_3_-N and PO_4_-P losses, and slope. The correlation coefficients of the nutrient losses, however, were higher with soil loss than with runoff.

## Discussion

### 4.1. Nutrient losses in runoff and eroded soil

#### Runoff and soil and nutrient losses

The annual amounts of runoff and soil loss were generally low for all treatments and sites, with the highest annual runoff and coefficient (20 L m^-2^) in T0 at site II and the highest annual soil loss (156 g m^-2^) in T0 at site III. These amounts were significantly lower than those reported for 2011 at the same sites [30] and at other locations in the watershed [2]. The low runoff and soil loss were due to the short rainy season, with rains of low erosivity during the season. The smallest runoff and largest erosion rates at site III can be attributed to the high soil erodibility associated to the higher silt content in this site than in the others [50].

The nutrient concentrations in the runoff (2.20–4.83 mg L^-1^ NO_3_-N and 0.02–0.07 mg L^-1^ PO_4_-P) and eroded soil (1.30–8.51 mg g^-1^ NO_3_-N and 5.27–18.8 mg g^-1^ PO_4_-P) were higher than those reported at the plot level in Burkina [5] and at the watershed level in Cabo Verde [39].

The amounts of NO_3_-N (0–18.9 kg ha^-1^) lost in runoff and eroded soil, particularly for site III, may have been higher than those in other hilly areas [22, 41], but total PO_4_-P (0–0.4 kg ha^-1^) losses were in similar ranges. All supplemented treatments (T1-T3, but particularly T3) significantly reduced or eliminated runoff and soil loss and the amounts of NO_3_-N and PO_4_-P lost in both runoff and sediments. Similar findings have been reported in Burkina Faso [5], Pakistan [22], and China [51, 52]. Indeed, runoff and water erosion can transport nutrients from the field either dissolved in solution or associated with soil particles, reducing the amount of nutrients available to support crop production [53–55].

The absence of a significant effect of the supplemented treatments on nutrient concentrations in both the runoff and sediments, and the positive effect of the treatments in reducing nutrient losses, indicated that the loss of nutrients in each treatment was mainly a function of the amount of runoff and soil loss, as previously reported [5, 22, 56], and was not dependent on the nutrient concentration of the eroded soil and runoff water [32, 57]. This dependence can also account for the large nutrient losses in the control plot, which lost the most soil, and the small nutrient losses in T3, which lost the least amount of soil.

Our results indicated that most of the NO_3_-N was lost in the runoff and most of the PO_4_-P was lost in the eroded soil, as also reported by other studies [5, 22, 52]. The higher PO_4_-P losses in the sediments in T1 (without hedges) compared with T2 and T3 (with hedges) indicated that the pigeon-pea hedges contributed to the lower PO_4_-P losses in the sediments. The effectiveness of all supplemented treatments to significantly reduce nutrient losses can be attributed to the organic amendments (compost or animal or green manure) applied to the plots, because their incorporation into the soil can improve aggregate stability and thus reduce NO_3_-N and PO_4_-P losses, as previously reported [5]. Mulch, as in the T3 plots, can protect soil against the impact of raindrops, decrease runoff velocity, improve the infiltration capacity of the soil, and thus control erosion and nutrient loss [3, 58, 59].

#### Nutrient inputs versus losses

Nutrient inputs in the supplemented treatments were higher than the losses, but the losses in T0 that received no inputs were higher than those in the supplemented treatments, indicating that the nutrients were lost from the original soil, as previously reported [22]. The partial nutrient balance was positive for all supplemented treatments at all sites, but the balance for the traditional system (T0) may have been low, particularly at sites II and III, which in the medium to long term could lead to nutrient depletion, crop nutrient deficiency, and lower yields. Despite the relatively low annual nutrient losses, the combination of a technique to control soil erosion with an organic amendment will contribute to better long-term sustainable land management, because these techniques will decrease soil loss, provide nutrients for better crop productivity, maintain soil fertility, and prevent nutrient depletion [60, 61].

### 4.2. Effect of the treatments on crop growth and yield

#### Crop yields and biomass

The responses of dryland maize yield to the treatments were clearly inconsistent due to the irregularity of the rainfall, which affected the crop at crucial stages of its development, as has also been reported in earlier studies in Cabo Verde [2, 38, 41]. The short and poorly distributed rains during the growing season also contributed to the overall low maize yield at the three sites.

The plots containing pigeon-pea hedges (T2 and T3, at sites II and III) produced a shading effect on the maize plants, hindering the development and yield of the maize after the complete development of the hedges. The yields at site I, which did not contain pigeon-pea hedges, supported this finding. The effect of shading may have also masked the performance of the combined treatments, suggesting that the hedges were spaced too closely and/or that the pigeon-pea plants needed to be managed to eliminate the shading on the maize crop. Our results corroborated those in another study with hedges of *L*. *leucocephala* in Burkina Faso [3]. Despite the positive effects of T2 and T3 on maize yield in some cases, the efficiency of the pigeon-pea hedges and mulch on maize yield was not conclusive for the cropping season studied. The ability of mulch combined with *L*. *leucocephala* hedges to improve the use of water resources by grain crops in semiarid regions has been questioned [2].

The high performance of T1, which consistently increased crop yield at all sites, showed the positive effect of both manure and compost combined with a soil surfactant, even though the plant nutrient content did not reach the sufficiency level. Thus, T1 can be considered the best treatment for flatter areas with deeper soils taking mainly crop yield into consideration however, on steep hillsides with shallow soils T3 will be the best treatment in order to prevent further land degradation due to runoff, soil erosion and nutrient loss. Given the efficiency of T3 in reducing runoff, erosion and nutrient loss (Table 7), it is the best treatment for the steep hillsides to guarantee the sustainability of soil management, as long as the pigeon-pea hedges are well managed to avoid shading.

Earlier studies in Cabo Verde [62, 63] reported a significant increase in maize yield with the application of animal manure and a positive effect of *Leucaena* prunings as an N source for dryland maize production. In the absence of inorganic N fertiliser, however, only very high doses (6 t ha^-1^) of *Leucaena* prunings significantly increased yield. Other studies [64, 65] have also reported positive effects of *Leucaena* leaf biomass on the N economy and productivity of maize.

The availability of *Leucaena* biomass in Cabo Verde depends on the regularity of rainfall, so large quantities of material may not be available for green manuring. Animal manure is also not plentiful, so composting crop residues with animal and green manures would be a more sustainable solution for supplying nutrients to dryland maize.

The yield of pigeon-peas from the hedges was not included in the yield analysis. The crop, though, is high yielding, and both green and dry grains have high nutritional and economic value and are well appreciated as human food. If pigeon-pea biomass from the hedges (T2 and T3 at sites II and II) had been included, our biomass values would have been significantly higher for these treatments. Pigeon-pea biomass would be an added value and incentive for the farmers because it is widely used for animal feed [44]. Pigeon-peas are a leguminous, high-yielding crop (5–12 t ha^-1^ dry biomass) with a relatively high N content (24–29 g kg^-1^), contributing to improve N budget when incorporated into maize-based cropping systems [66]. Pigeon-pea prunings can also be used as soil cover to protect the soil from erosion and to improve soil fertility.

#### Plant nutrient status

The N content of the plant tissues was below the sufficiency level [67] in all treatments at all sites, but the P content was at or above the sufficiency level in all treatments at all sites. Maize is a nutrient-demanding staple crop, so symptoms of deficiency appear and grain yield and quality decline when N levels drop below the sufficiency range [68]. N is a limiting nutrient in dryland maize in Cabo Verde [69], and deficiency symptoms are widespread on the hillsides. In fact, symptoms of N deficiency were registered at all sites in both seasons.

The absence of a significant response to the application of the organic nutrient sources may have been due to the low N content of the compost (22 g N kg^-1^), animal manure (23 g N kg^-1^), and green manure (36 g N kg^-1^) applied to the soil, in which case higher rates of organic amendments would be required to supply adequate nutrients to the maize plants. For soils low in N and P, application rates of 100 and 40 kg ha^-1^ of N and P, respectively, have been reported for optimum yields of local varieties of maize [70, 71]. Another possibility for the absence of a significant response could be a slow rate of N mineralisation in the amendments, particularly the manure, which would reduce the amount of N available to the crops during the cropping season [72]. Increasing the amount of organic material, particularly the green and animal manures, to satisfy the N and P requirements may not be feasible due to low availability and to the poor economic conditions of the farmers.

### 4.3. Relationships among the various parameters

The treatment component was responsible for 43% of the variance in the data and was strongly associated with most parameters related to the nutrient losses in the runoff and sediments, confirming the positive effect of the techniques of land management on nutrient losses. Soil loss increased with increasing runoff, and nutrient losses increased with the amounts of runoff and soil losses, indicating that the factors influencing runoff and soil loss (i.e. soil cover, vegetation runoff hedges, rain erosivity, and slope), as previously reported [26, 30, 31], also influenced the nutrient losses [5, 22]. These findings could account for the high effectiveness of the treatment containing mulch, pigeon-pea hedges, and an organic amendment (T3) in reducing nutrient losses associated with runoff and erosion. The stronger correlation between nutrient losses and soil loss compared to runoff indicated higher NO_3_-N and PO_4_-P losses in the eroded sediments at all sites.

Maize and biomass yields, the second most important component and responsible for 30% of the variance in the data, were not significantly correlated with treatment application or the nutrient losses but were positively correlated with soil moisture and rainfall. This lack of a generalised effect of the supplemented treatments on both maize yield and biomass was highlighted in section 4.2 and was due mainly to the variability of rainfall and the low soil moistures in some of the treatments during critical periods of the cropping season. For example, T2 and T3 at site II contained pigeon-peas and thus had higher plant biomasses than did T0 and T1, and the competition for water was greater, which together with the low water-retention capacity of the sandy loam soil could account for the low moisture content. At site III, however, as the plants reached full development and rainfall and moisture was more abundant, T2 and T3 registered higher soil-moisture contents than did T0 and T1, despite the high use of water by the abundant biomass, perhaps due to the high water-holding capacity of the soil.

The absence of a significant correlation between the crop parameters (maize, bean, and biomass yields) and the nutrient losses may have been due to the low losses, not affecting yield or plant development. The strong correlation between the nutrient losses and rainfall at each site, though, was an indication of the importance of a regularly distributed rainfall for providing moisture to the crops throughout the growing season; soil moisture was responsible for 16% of the variability in the data. The difference in soil-moisture content between the sites was due to the different soil textures, which influenced infiltration and the capacity of the soil to retain water in the order silty clay loam>loam>sandy loam.

## Conclusions

This study evaluated the effects of selected strategies of land management on nutrient losses in runoff and sediments and on crop yield. The following conclusions can be drawn:

- The combination of an organic amendment with soil surfactant (T1) could be considered the best treatment for flatter areas with deeper soils less affected by soil erosion and setting the focus on increased crop yield.
- The combination of crop-residue mulch with organic amendment and runoff hedges (T3) could be the best treatment to prevent further land degradation due to runoff, soil erosion and nutrient loss on steep hillsides with shallower soils, as long as the pigeon-pea hedges are well managed to guarantee higher maize yield.
- Further research should focus on long-term field trials to include a wider range of rainfall conditions and should test different rates of organic amendments. This study aimed to help smallholder subsistence farmers, so an evaluation of the cost-effectiveness of the selected technologies will be crucial in establishing sustainable options under semiarid hillside conditions and in determining their biophysical and socio-economic applicability at a wider scale.
- Effective farmer involvement through the establishment of demonstration plots and farmer education and awareness of the need to prevent the degradation of soil fertility for sustainable dryland yields will be essential to foster the adoption and successful implementation of the selected strategies.
- Strategies of sustainable land management that increase the use of rainwater, prevent the degradation of soil fertility, and potentially increase sustainable dryland yields should be promoted and implemented on the semiarid hillsides in Cabo Verde.
